# Supplementary material for: Gene content evolution in the arthropods
Source: Genome Biol. 2020 Jan 23;21:15. doi: 10.1186/s13059-019-1925-7 (PMC6977273; doi:10.1186/s13059-019-1925-7)
Supplement: Supplementary file 3 — Review history. [file 13059_2019_1925_MOESM3_ESM.docx]

Review History

**First round of review**

**Reviewer 1**

**Are you able to assess all statistics in the manuscript, including the appropriateness of statistical tests used?** There are no statistics in the manuscript.

**Comments to author:**

The authors present the largest set of arthropod genomes ever by combining existing 48 genomes on top of their 28 newly sequenced genomes.
Their extensive sequencing and analyses as a set of biological resources justifies an immediate publication.

There are no major issues scientifically speaking.

minor comments:

>....xenobiotic defense (cytochrome P450s, sulfotransferases), digestion (peptidases), chitin exoskeleton structure and
>metabolism, multiple zinc finger transcription factor types, HSP20 domain stress response, fatty
>acid metabolism, chemosensation, and ecdysteroid (molting hormone) metabolism (Table S15).

It makes sense and this reviewer wonder if there are any reproductive system related rapid/big gene family changes(Not asking to do
any more analyses, but readers may wonder).

>GO-term analysis of these 147 gene families recovered
>multiple key functions, including cuticle and cuticle development (suggesting changes in
>exoskeleton development), visual learning and behavior, pheromone and odorant binding
>(suggesting the ability to sense in terrestrial/aerial environments rather than aquatic), ion
>transport, neuronal activity, larval behavior, imaginal disc development, and wing
>morphogenesis.

I do not know the biology of arthropods, but how about immune system related gene family changes(expansion and contraction) across the Arthropoda phylum.

>The majority of domain arrangements (48% of all observable events) were
>formed by a fusion of two ancestral arrangements, while the fission of an existing arrangement
>into two new arrangements accounts for 14% of all changes. Interestingly, 37% of observed
>changes can be explained by losses (either as part of an arrangement (14%) or the complete loss
>of a domain in a proteome (23%)), while emergence of a novel protein domain is a very rare
>event, comprising only 1% of total events.

I doubt there is any 'complete' loss in the proteome as those could be undetected as pseudogenes by usual homology searches.
If there are really 'complete' protein domain disapprearance in any phylo-branch, I would be surprised.
(I don't want to go into search/alignment algorithm sensitivity and thresholds issues, but by using distant homology
search using HMM or iterative intermedidate sequence search such as PSI-blast, the distant homolog detection rate
could change by 2 folds changing the analyses landscape.

This dramatic bias could be the usual trend that it is easy to 'fail to find distant homologs' that drifted away while we can detect
fused protein domains more easily. (not critically changing the science, but it could be considered in all the interpretation)

Also, it used to be known (I am not sure if it still holds) that as organisms evolve to be more complex, it is general that protein domain
fusion events become more frequent and the overall protein size becomes larger (a general trend point)


>We observe high concordance between rates of gene family dynamics and protein domain
>rearrangement (Figs. 4 & S28).

This is expectedly interesting.

>we find a high rate of new protein domains in the subphylum Chelicerata, including a
>large number within Araneae associated with venom and silk production.

Are you sure that there are "new protein domains"? I persnally doubt it. It could be rapid changes on coiled coil loops etc
rather than actually a new domain.

The authors could have checked the domain sizes of the presumably ''new'' domains. Are they peptides as they are mostly
toxins?

>We also find the largest number of domain rearrangement
>events in B. germanica. The impressive capability of this cockroach to quickly adapt to changing
>environments could be linked to these numerous and rapid evolutionary changes

Are the authors suggesting that the changes were selection driven? Or some "adaptation" capability built in the roaches?
(if it is to do with undesired domestication of roaches by humans, authors suggest rapid changes happened in domesticated
plants and animals? Also, how fast (10,000s years)?

>Though we observe thousands of genomic changes across the arthropod phylogeny, they
>are mostly evenly distributed (Fig. 3D).

I wonder if the authors attempted to do genome SV analyses across the species at low level branches albeit the genetic distances can be too large to do them effectively.

>43.0 gains/losses per My

Genes cannot be really 'lost' completely usually and also, I doubt they can gain really new genes. I guess it means the gene families are not detectable as properly structured genes in the big pool of non-coding regions in a species?

Also, here, 43 'genes' or gene families? Proteins, domains, and genes usually form families(groups) in species (including pseudogenized ones).

>They are instead due mostly to single gene gains or losses in a large number of gene families.

OK. This makes sense. 43 genes/My, then.

>For example, terminal branches within the Hymenoptera have an accelerated rate of
>domain rearrangement, which coincides with the increased rate of gene gains and losses
>observed along those branches. This novel finding is surprising, given that these processes
>follow largely from different underlying genetic events.

This is not surprising at all (as expected).

Can you clarify what you have in mind in saying "these processes" follow largely from different underlying genetic events ?

Domain and gene duplication/fusion/loss etc are usally associated or linked to each other.


>Similarly, the hymenopteran lineages displaying the fastest rate of gene gain/loss in our analysis do not display higher rates of amino acid substitutions.

I find this one is rather surprising. It could mean that AA changes could be compensated by gain/loss and vice versa.(that makes sense, but it could have been co-related)


>We see an increased rate of gene family extinction along the ancestral nodes from the ancestor of the cockroach and termites and
>hemimetabolous insects to the ancestor of Lepidoptera and Diptera (Fig S20), suggesting evolution by gene loss12,42.

I would be careful on this interpretation. The supposedly ancestoral nodes could have been just one of thousands of other ancestoral species long time ago(mostly exinct now).
Therefore, gene loss must interpreted very carefully. Also, all phylogenetic trees are mathematically flawed as we do not have extinct species in millions of years and  trees do not exist and all our trees are complex networks. Therefore, it is possible that some roach branches did not evolve by gene loss but having a different biopath sets that have been just one type of much longer history of arthropods. Also, as I pointed out earlier, detecting very distant homologs has a big limit. I think almost all genomics study is affected by this bias of undetectable very distant homologs (a mathematically challenging problem).

>The current study was unable to address the evolutionary non-coding sequences such as enhancers, promoters and small and other noncoding
>RNAs underlying these networks due to the lack of sequence conservation over large
>evolutionary distances, however our results underscore their evolutionary importance.

This is also an aspect that gene gain/loss can be simplistic sometimes in explaining functional evolution.

**Authors Response**

**Point-by-point responses to the reviewers’ comments:**

minor comments: (1) ....xenobiotic defense (cytochrome P450s, sulfotransferases), digestion (peptidases), chitin exoskeleton structure and metabolism, multiple zinc finger transcription factor types, HSP20 domain stress response, fatty acid metabolism, chemosensation, and ecdysteroid (molting hormone) metabolism (Table S15). It makes sense and this reviewer wonder if there are any reproductive system related rapid/big gene family changes (Not asking to do any more analyses, but readers may wonder).

**Reply:** Sorry - but they are not in the top 30. To be honest, I (SR) was hoping for something exciting like that linking to speciation in this most specious of phyla, but it didn’t pop out. Another possibility is that genes encoding proteins involved in reproduction that might be on the forefront of possible speciation mechanisms such as sperm competition - might be changing too fast to be picked up as gene families and instead all show up as genes of unknown function unique to a species? - We agree that this is an interesting area for further research. Unfortunately, we can’t say anything rigorous for the reader at this point.

(2) GO-term analysis of these 147 gene families recovered multiple key functions, including cuticle and cuticle development (suggesting changes in exoskeleton development), visual learning and behavior, pheromone and odorant binding (suggesting the ability to sense in terrestrial/aerial environments rather than aquatic), ion transport, neuronal activity, larval behavior, imaginal disc development, and wing morphogenesis. I do not know the biology of arthropods, but how about immune system related gene family changes (expansion and contraction) across the Arthropoda phylum.

**Reply:** Interestingly we find no GO immune terms enriched among gene gain and loss events in any node. It will take additional work to better understand the gain and loss of known immune genes across the Arthropoda.

(3) The majority of domain arrangements (48% of all observable events) were formed by a fusion of two ancestral arrangements, while the fission of an existing arrangement into two new arrangements accounts for 14% of all changes. Interestingly, 37% of observed changes can be explained by losses (either as part of an arrangement (14%) or the complete loss of a domain in a proteome (23%)), while emergence of a novel protein domain is a very rare event, comprising only 1% of total events.

I doubt there is any 'complete' loss in the proteome as those could be undetected as pseudogenes by usual homology searches. If there are really 'complete' protein domain disapprearance in any phylo-branch, I would be surprised. (I don't want to go into search/alignment algorithm sensitivity and thresholds issues, but by using distant homology search using HMM or iterative intermedidate sequence search such as PSI-blast, the distant homolog detection rate could change by 2 folds changing the analyses landscape. This dramatic bias could be the usual trend that it is easy to 'fail to find distant homologs' that drifted away while we can detect fused protein domains more easily. (not critically changing the science, but it could be considered in all the interpretation) Also, it used to be known (I am not sure if it still holds) that as organisms evolve to be more complex, it is general that protein domain fusion events become more frequent and the overall protein size becomes larger (a general trend point)

**Reply:** On the last point of protein domain fusion events becoming more frequent, we agree that this is largely true and speaks for fusion being more frequent than fission. see: Moore AD et al Biochim Biophys Acta. 2013 doi: 10.1016/j.bbapap.2013.01.007, Kummerfeld S + Teichmann S Trends Genetics 2005, DOI: 10.1016/j.tig.2004.11.007. On the subject of ‘Loss’, ‘Loss’ is used here as a term from a functional perspective. We consider the coding content of the genome in this study and therefore any mutation that leads to a gene knockout, pseudogenisation etc. would remove the corresponding gene product as a functional unit from the protein repertoire of the related organism/lineage. The same way introduced stop codons and other mutational events can shorten a gene and lead to the loss of sub-genic units/domains as functional parts of the protein repertoire.

By usage of the term ‘loss’ we do not want to state that the related sequences are all removed completely from the genome, but that they do not play a functional role anymore and are often on a sequence level not detectable as homolog sequence parts, since the selection pressure on the non-coding sequences is removed and mutations can accumulate randomly, especially over the 100’s of millions of years investigated here.

Regarding the problem of homology detection and algorithm sensitivity we are aware of the problem and this is a valid point. However, we use HMMs for the detection of protein domains and have implemented therefore already a more sensitive method than standard sequence similarity-based methods. Additionally, the conducted jack-knife test can reveal error ranges for the different observed event types, which can result from non-detection of certain genes/domains.

We are therefore confident that the mentioned problems are addressed with the currently available state-of-the-art methods and that the majority of losses are indeed (functional) losses as defined above.

We observe high concordance between rates of gene family dynamics and protein domain rearrangement (Figs. 4 & S28). This is expectedly interesting. (5) we find a high rate of new protein domains in the subphylum Chelicerata, including a large number within Araneae associated with venom and silk production. Are you sure that there are "new protein domains"? I personally doubt it. It could be rapid changes on coiled coil loops etc rather than actually a new domain.

**Reply:** As described above for the definition of ‘loss’ this is likely a problem of contrasting definitions in different fields and a matter of perspective. We decided to go with the terms ‘new/novel’ or ‘emerged’ domains and genes, since this is widely used in the same context. The meaning of the term “new protein domain” here is the emergence of a domain at a certain node in the phylogenetic tree that did not exist before and provides potentially a previously not existing function. How novel domains or *de novo* genes can arise and through which molecular mechanisms existing non-coding sequence fragments can become functional / gain transcription or regulatory elements is out of the scope of this study, but has been investigated on domain and gene levels (e.g. Origins and structural properties of novel and de novo protein domains during insect evolution. 2018. Klasberg, Bitard-Feidel, Callebaut, Bornberg- Bauer. FEBS J.; Becoming a de novo gene. Bornberg-Bauer, E. and Heames, B. Nature Ecology & Evolution, 2019). All domains described in this study are from the Pfam database and therefore need to be conserved over some time in a specific clade or lineage (they are not new in the sense that they appeared recently or in single species, but are new at a specified node in the phylogenetic tree, providing new functionality for the sub-clade branching from that node.

(6) The authors could have checked the domain sizes of the presumably ''new'' domains. Are they peptides as they are mostly toxins?

**Reply:** Many of the toxins are indeed peptides and have an average size of around 30-60 amino acids. However, this is not an exclusive property of all new domains, as there are other emerged domains than toxins (the mentioned domains in spiders are 80-170 aa long) and also some toxin domains are larger, as for example the CagA exotoxin (PF03507) mentioned for the bark scorpion has a length of 190 aa (HMM model length, average domain length in sequences ~140 aa). With the provided Pfam-IDs the length of every domain we found to be emerged in the analysis can be checked on the Pfam homepage.

(7) We also find the largest number of domain rearrangement events in B. germanica. The impressive capability of this cockroach to quickly adapt to changing environments could be linked to these numerous and rapid evolutionary changes Are the authors suggesting that the changes were selection driven? Or some "adaptation" capability built in the roaches? (if it is to do with undesired domestication of roaches by humans, authors suggest rapid changes happened in domesticated plants and animals? Also, how fast (10,000s years)?

**Reply:** We thank the reviewer for catching this less than rigorous speculation. We have toned down the sentence to merely state that this interesting and needs further investigation. - it now reads: “The impressive capability of this cockroach to survive many environments and it’s social behavior could be linked to these numerous and rapid evolutionary changes at the genomic level and warrants more detailed investigation.”

(7) Though we observe thousands of genomic changes across the arthropod phylogeny, they are mostly evenly distributed (Fig. 3D). I wonder if the authors attempted to do genome SV analyses across the species at low level branches albeit the genetic distances can be too large to do them effectively.

**Reply:** As the reviewer no doubt suspected we did not. Unfortunately, this is beyond both the quality of the current assemblies and the long evolutionary distances. Looking towards the future, as taxa are filled in with more closely related genomes, and more long read genome assemblies become available this will become feasible.

(8) 43.0 gains/losses per My Genes cannot be really 'lost' completely usually and also, I doubt they can gain really new genes. I guess it means the gene families are not detectable as properly structured genes in the big pool of non-coding regions in a species?

**Reply:** As described above gains and losses are defined here according to the functional repertoire available to an organism and the corresponding sequences of former genes could still exist in some form in the non-coding regions of a species.

Also, here, 43 ‘genes’ or gene families? Proteins, domains, and genes usually form families(groups) in species (including pseudogenized ones). They are instead due mostly to single gene gains or losses in a large number of gene families. OK. This makes sense. 43 genes/My, then. For example, terminal branches within the Hymenoptera have an accelerated rate of domain rearrangement, which coincides with the increased rate of gene gains and losses observed along those branches. This novel finding is surprising, given that these processes follow largely from different underlying genetic events. This is not surprising at all (as expected). (9) Can you clarify what you have in mind in saying "these processes" follow largely from different underlying genetic events? Domain and gene duplication/fusion/loss etc are usally associated or linked to each other.

**Reply:** We have added a reference to point the reader to a review of the differences in these processes. The sentence now reads: “This novel finding is surprising, given that these processes follow largely from different underlying genetic events (See Klasberg et Al [X] for discussion of these processes)”

Similarly, the hymenopteran lineages displaying the fastest rate of gene gain/loss in our analysis do not display higher rates of amino acid substitutions. I find this one is rather surprising. It could mean that AA changes could be compensated by gain/loss and vice versa.(that makes sense, but it could have been co-related) We see an increased rate of gene family extinction along the ancestral nodes from the ancestor of the cockroach and termites and hemimetabolous insects to the ancestor of Lepidoptera and Diptera (Fig S20), suggesting evolution by gene loss12,42. I would be careful on this interpretation. The supposedly ancestoral nodes could have been just one of thousands of other ancestoral species long time ago(mostly exinct now). Therefore, gene loss must interpreted very carefully. Also, all phylogenetic trees are mathematically flawed as we do not have extinct species in millions of years and trees do not exist and all our trees are complex networks. Therefore, it is possible that some roach branches did not evolve by gene loss but having a different biopath sets that have been just one type of much longer history of arthropods. Also, as I pointed out earlier, detecting very distant homologs has a big limit. I think almost all genomics study is affected by this bias of undetectable very distant homologs (a mathematically challenging problem).

**Reply:** We agree and have toned down the sentence, so it now reads: “suggesting the possibility of evolution by gene loss 12,42”.

The current study was unable to address the evolutionary non-coding sequences such as enhancers, promoters and small and other noncoding RNAs underlying these networks due to the lack of sequence conservation over large evolutionary distances, however our results underscore their evolutionary importance. This is also an aspect that gene gain/loss can be simplistic sometimes in explaining functional evolution.

**Reply:** We fully agree - hence this caveat was added to remind the reader.

Once again, we would like to thank the reviewer for the time and effort put into reviewing this manuscript. It is very much appreciated.
